# Aging and Error Processing: Age Related Increase in the Variability of the Error-Negativity Is Not Accompanied by Increase in Response Variability

**DOI:** 10.1371/journal.pone.0017482

**Published:** 2011-02-28

**Authors:** Sven Hoffmann, Michael Falkenstein

**Affiliations:** Leibniz Research Centre for Working Environment and Human Factors, Dortmund, Germany; Cuban Neuroscience Center, Cuba

## Abstract

**Background:**

Several studies report an amplitude reduction of the error negativity (Ne or ERN), an event-related potential occurring after erroneous responses, in older participants. In earlier studies it was shown that the Ne can be explained by a single independent component. In the present study we aimed to investigate whether the Ne reduction usually found in older subjects is due to an altered component structure, i.e., a true alteration in response monitoring in older subjects.

**Methodology/Principal Findings:**

Two age groups conducted two tasks with different stimulus response mappings and task difficulty. Both groups received fully balanced speed or accuracy instructions and an individually adapted deadline in both tasks. Event-related potentials, Independent Component analysis of EEG-data and between trial variability of the Ne were combined with analysis of error rates, coefficients of variation of RT-data and ex-Gaussian fittings to reaction times. The Ne was examined by means of ICA and PCA, yielding a prominent independent component on error trials, the Ne-IC. The Ne-IC was smaller in the older than the younger subjects for both speed and accuracy instructions. Also, the Ne-IC contributed to a much lesser extent to the Ne in older than in younger subjects. RT distribution parameters were not related to Ne/ERP-variability.

**Conclusions/Significance:**

The results show a genuine reduction as well as a different component structure of the Ne in older compared to young subjects. This reduction is not reflected in behaviour, apart from a general slowing of older participants. Also, the Ne decline in the elderly is not due to speed accuracy trade-off. Hence, the results indicate that older subjects can compensate the reduction in control reflected in the reduced Ne, at least in simple tasks that induce reaction slips.

## Introduction

The monitoring, detection and processing of errors is crucial for efficient adaptation of behavior. In the last 20 years increasing evidence pointed to an adaptive system for the control and monitoring of (re-)actions. The first evidence for a neural correlate of such a system came from EEG studies. Errors in simple reaction choice tasks (“slips”) provoke a typical event-related potential (ERP): the “error negativity” (Ne, [1]) or “error-related negativity” (ERN, [2]).

The Ne reaches its (negative) maximum at fronto-central electrode sites at about 50–80 ms following an erroneous response. Its generators have been located reliably in the anterior cingulate cortex (ACC) [3], [4], [5].

The impact of aging on performance or error monitoring has been addressed with several studies [6], [7], [8]. It has repeatedly been shown that the Ne is attenuated in older subjects [6], [7], [8], [9], [10], [11]. However, this was not the case in all studies: it has been shown that the age effect on the Ne was affected by performance or mediated by learning effects [12], [13]. Also, there exists evidence, that in learning tasks, the Ne of older subjects is not attenuated if both groups are matched by accuracy [13].

Recent studies have reported consequences for behavioral adaption following erroneous responses (e.g. error rate, post-error slowing) in elderly compared to younger subjects (e.g., [10], [14]).

Up to now the source of the often reported decline in the amplitude of the Ne is not clear: is it due to a true age related decline in the ability to monitor responses and errors, or is the reported decline a consequence of the utilized tasks? It can be shown, that the Ne is attenuated in tasks which are more difficult or have a weaker stimulus response mapping than for example a flanker task (e.g., [3], [7]). It might be, that elderly can compensate the declined activity by recruiting additional resources. Also, it might be that the declined amplitude in elderly is due to a smearing of the Ne on the single trial level, i.e. the latency of the Ne varies from trial to trial, and thus the average Ne declines. However, in this case the question would arise what the function of the Ne is in general.

The present study aims to test whether the age-related decline in Ne-amplitude is due to a differential component structure underlying the observed Ne and whether the Ne reduction is linked to behavioural consequences. In earlier studies it was shown that in young subjects one component (termed Ne-IC in the following) can explain most of the variance in the Ne [4 5,15,16]. We aimed to test whether this is also true for older subjects, and whether this component is linked to behavioural parameters, such as error rate, response speed, and RT distribution.

For this purpose behavioural data were not only analyzed by means of average response times and error rates, but also ex-Gaussian functions were fitted to the response time data in order to test whether the Ne is linked to behavioural variability in older subjects. Ex-Gaussians were fitted, since the problem with standard comparisons of average response times is that between group differences with respect to mean reaction times might not be a result of the shift of both distributions relative to each other, but rather due to an increase of skew for older adults. The fitting of an ex-Gaussian distribution circumvents such problems of interpretation of measures of central tendency (aka mean) [17], [18], [19]. Briefly described, the ex-Gaussian distribution represents the convolution of two functions: a normal (Gaussian) function and an exponential function. The fitting of the ex-Gaussian distribution yields three parameters: the µ (mu)-, σ (sigma)-, and τ (tau)-parameter. Mu represents the mean of the Gaussian function and reflects average performance, sigma represents the standard deviation of the Gaussian function and reflects variability of performance, and tau is the mean and standard deviation of the exponential part of the function and reflects extreme values in performance.

It is a common result, that reaction times of older people are more variable than those of younger ones. Thus, the question arises, if this increased RT variability is linked to differences in information processing, e.g. the effectiveness of cognitive control.

One interpretation is, that the response times of older are more variable because they are slower (e.g., [20], [21]). However, reaction time distributions of older are typically associated with larger tau values (indicating larger skew of the distributions), which lead to the hypothesis that older show increases in lapses of attention, failures of inhibition and, most importantly for the present study, fluctuations in the efficiency of cognitive control (e.g., [22], [23], [24], [25], [26]). However, for subtle processes related to cognitive control reaction times might not be a sensitive measure, thus in the present study behavioral analyses are combined with the analysis of the EEG.

One crucial factor influencing the age-effect on the Ne may be a priori age differences in the perceived task difficulty of the stimulus response mapping. It is known that task difficulty has an impact on the Ne, i.e. it is attenuated in difficult tasks [7]. Hence, it might be that elderly experience the same task as being more difficult than young subjects do. Therefore two tasks with different stimulus response mapping were conducted. The first task was a flanker task, and the second one a mental rotation task. The conduction of two different tasks, with the flanker task as benchmark, would allow to investigate whether the Ne generalizes to more complex tasks, i.e. more difficult tasks within subjects. In summary, we were interested to investigate how the Ne is influenced by task-specific variance and how this is modulated by age. The Ne of a standard flanker task serves as a benchmark against the Ne from a task with much weaker stimulus response mapping, i.e. a mental rotation task. These comparisons could yield further information about the sensitivity of the Ne to different levels of executive function demanded by both tasks. In addition, analyzing individual differences in the error monitoring response between and within age groups could provide more insight into the functional significance of the Ne in general.

## Results

### Behavioural data

On average older participants responded more slowly than younger ones [F(1,34) = 8.18, p<.01, η^2^ = .19]. The reaction times (RTs) were faster in the flanker compared to the rotation task [F(1,34) = 137.52, p = .01, η^2^ = .8] and in erroneous compared to correct responses [F(1,34) = 138.12, p<.001, η^2^ = .8]. Also, there was a significant interaction of task and response [F(1,34) = 74.69, p<.001, η^2^ = .69], indicating that the difference between erroneous and correct response were greater for the flanker task than for the rotation task. The corresponding descriptive statistics, i.e. mean RT and standard deviations are provided in Table 1.

**Table 1:** Reaction time data of both tasks and age groups.

Task	Response	RT (mean)	RT (sd)	µ	σ	τ	
***Flanker***	Error	244	23	215	19	29	
	Correct	317	24	265	41	51	
***Rotation***	Error	456	106	378	59	62	
	Correct	464	81	354	52	95	
***Flanker [old]***	Error	292	21	247	26	44	
	Correct	364	20	305	50	58	
***Rotation [old]***	Error	509	120	389	54	89	
	Correct	522	93	427	70	75	

Descriptive statistics by means of average reaction time[RT(mean)], standard deviation [RT(sd)] and ex-Gaussian parameters [µ,σ,τ].

In order to test whether the RT difference is due to a different RT variability of both age groups, coefficients of variation were calculated yielding no significant difference between both groups. In general the error rate was higher for the rotation task [F(1,34) =  7.63, p<.01, η^2^ = .18]. The error rates did not differ between older and young participants.

Concerning the ex-Gaussian RT-data fit the ANOVAs revealed that the parameters were not all in line with the standard RT-data analysis. Older subjects had a lower µ-parameter [F(1,34) = 4.89, p = .03, η^2^ = .12] whereas the other parameters did not differ significantly between groups, which means that the older subjects did not show a higher RT variability than the younger ones, nor did they produce more outliers. However, in general, they responded more slowly. With respect to the task effect, the µ-, σ-, and τ-parameters were lower for the flanker task than for the rotation task [F (1,34) = 83.53, p<.001, η^2^ = .71; F (1,34) = 35.91, p<.001, η^2^ = .51; F(1,34) = 37.80, p<.001, η^2^ = .52]. Thus, participants responded faster in the flanker task compared to the rotation task. Additionally, RTs were less variable [σ] in the flanker task and contained of fewer outliers [τ] (i.e. slower responses) in the flanker task compared to the rotation task. This was also true for the response effect: µ-, σ-, and τ-parameters were smaller for erroneous responses compared to correct responses [F (1,34) = 62.38, p<.001, η^2^ = .65; F (1,34) = 45.74, p<.001, η^2^ = .57; F(1,34) = 25.17, p<.001, η^2^ = .42]. Thus, on average, RTs of erroneous responses were shorter, less variable, and contained fewer outliers than RTs for correct responses.

Additionally, the interaction between age group and response became significant for all three parameters [F (1,34) = 22.68, p<.001, η^2^ = .40; F(1,34) = 9.62, p = .004, η^2^ = .22; F(1,34) = 2.68, p<.001, η^2^ = .37]. Hence, RT differences between incorrect and correct responses were not as large for the younger subjects compared to the older subjects. But this is due to the three way interaction of group, task and response [below]: in the rotation task, the RTs of young subjects did not differ substantially between erroneous and correct responses, whereas for the old subjects there remained a marginal difference: the incorrect responses were faster than the correct ones.

With respect to the task and response interaction the µ and σ parameters became significant [F(1,34) = 18.64, p<.001, η^2^ = .35; F(1,34) = 13.34, p<.001, η^2^ = .28]. Thus, reaction time varied as a function of task and response type: in the flanker task erroneous responses were faster compared to correct responses, but this was not the case in the rotation task. This was also true for the σ-parameter: erroneous responses showed more variability in the flanker task, compared to the rotation task (see table 1).

Finally, the three-way interaction between age group, task and response became significant for µ and τ [F(1,34) = 4.98, p = .03, η^2^ = .13; F(1,34) = 4.22, p = .048, η^2^ = .11].

Table 1 summarizes the mean reaction times and the parameter estimates.

All single-subjects fittings showed no significant differences between ex-Gaussian fits and empirical probability density functions.

With respect to the coefficient of variation of response times older and younger subjects did not differ significantly [F(1,34)  = 0.4759, p = .49, η^2^ = .01]. This means that the mean RTs of both groups are valid for comparison, since the mean is not distorted by different variances in both groups. Also, the comparable coefficient of variation of both groups supports the results of the ex-Gaussian fit where σ- and τ-parameters did not differ significantly.

However, the RTs in the rotation task were more variable than the RTs in the flanker task [F(1,34) = 6.59, p = .014, η^2^ = .16]. Also, RTs of correct responses were longer than RTs of erroneous responses [F(1,34) = 68.28, p<.001, η^2^ = .67]. The significant interaction of task and response indicated that in the flanker task the RT variability was higher for correct responses compared to erroneous responses [F(1,34) = 25.78, p<.001, η^2^ = .43].

### EEG data

**Figure 1:**
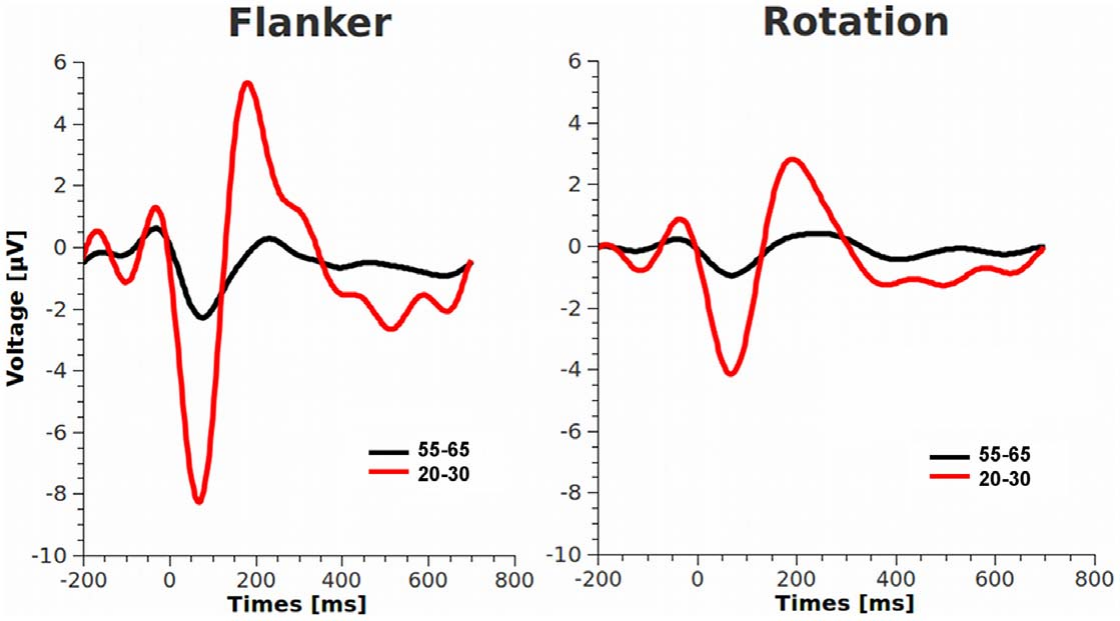
Error-related negativity (response-related) for both groups and tasks parameterized at FCz. Red: Young subjects, black: older subjects. Zero indicates button press.

With respect to the electrophysiological analysis the results show that the amplitude of the Ne is attenuated in older subjects [F(1,34) = 26.06, p<.001, η^2^ = .44] [Figure 1] and in the rotation task compared to the flanker task [F(1,34) = 31.97, p<.001, η^2^ = .48].

**Figure 2:**
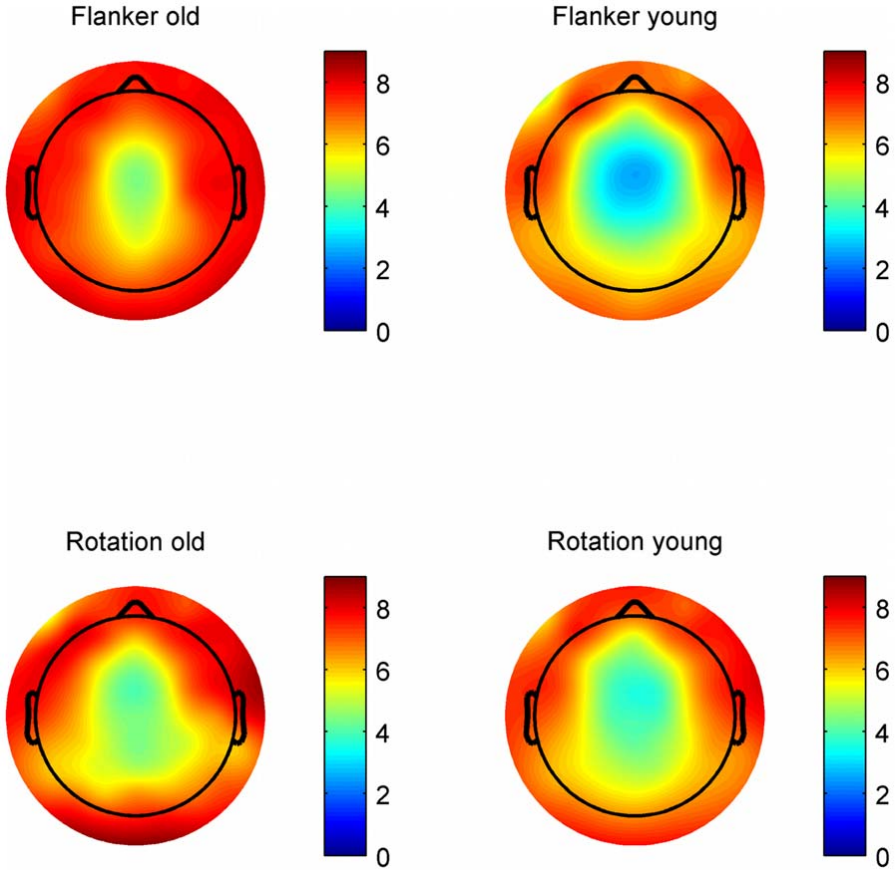
Topographic distribution of the average number of principle components accounting for 90% of variance. Estimation of the variability between trials in the Ne-time window for both tasks. Blue indicates less variability between trials; red indicates more variability between trials. Beside each topography a colour legend is provided indicating the number of principle components (PCs) explaining at least 90% of variance between trials. For example having a look at the topography of the young subjects in the flanker task, one can see that at FCz about three principle components explain at least 90% of variance between trials. In older participants (left) about five principle components explain at least 90% of variance between trials. Note that in general the signal is least variable at fronto-central positions and reflects the typical Ne-topography.

With respect to the estimation of the trial-to-trial variability of the Ne at FCz by means of PCA the results show that the Ne of older participants showed more variability between trials than the Ne of younger subjects [F(1,34) = 28.55, p<.001, η^2^ = .46] and in the rotation task compared to the flanker task [F(1,34) = 11.3, p<.01, η^2^ = .25]: In younger subjects, fewer principle components account for 90% of the variance between the trials than for older subjects. This was true for the flanker task as well as for the rotation task. Figure 2 shows the scalp distributions of the variability, which was calculated by estimating the number of components which explain at least 90% of variance for the erroneous trials with respect to the Ne-time window.

In addition, fewer elderly participants showed a typical Ne-IC (11/16 vs. 18/20). However, an exact Fisher-Yates test revealed that this difference was not significant (p = .20, odds ratio = .25).

**Figure 3:**
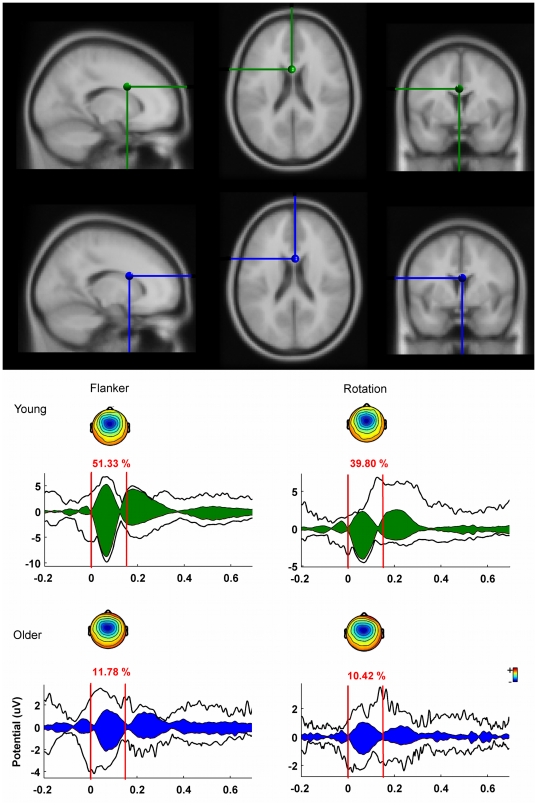
Dipole localizations and contributions of the Ne-IC cluster to the grand average ERP. Upper panel: Dipole localizations of the Ne-IC cluster of the young group [Tal(x,y,z) = 2,10, 19; residual variance = 7.11%] and older subjects [Tal(x,y,z) = −1,10, 26; residual variance  = 7.1%].(young = green, old = blue). Lower panel: Contributions of the Ne-IC clusters of each group in the time-window from 0–150 ms following response. *Flanker task:* The Ne-IC of young subjects cluster accounted for about 51% of variation in the time window 0–150 ms following erroneous response. The Ne-IC of older participants accounted for about 11% of variation in the Ne time window. *Rotation task:* Here, Ne-IC cluster of older participants accounted for fewer percent of variation compared to younger subjects, too [39.8%<10.42%].

In older subjects the Ne-IC cluster explained less percent of variation [11%<51%] in the Ne time-window compared to the young group in the flanker task [Figure 3]. In the rotation task the Ne-IC cluster also explained less percent of variation [10%<39%] for older compared to younger subjects[Figure 3]. The explained percent of variance was estimated by including only subjects which showed a Ne-IC.

## Discussion

The present study does not only show that the Ne is not fully comparable between older and younger subjects, but also that the often reported amplitude difference cannot be explained solely by a higher response variability of older people. Though participants of both groups differed with respect to the Ne-Amplitude, they did not differ in error rate which was forced by an adaptive deadline. However, they did differ in average reaction time, indicating that older subjects do not perform “worse” in terms of errors, but they just respond slower. Thus, the difference between groups in Ne-amplitude is not due to speed-accuracy trade-off.

Furthermore, the RTs of older subjects were only longer on average but not more variable than RTs of younger subjects. Also, the standard RT analysis masked the interactions between group and response type (erroneous vs. correct response) and the interaction between group, task and response. Obviously, the tasks had quite a differential impact on the behavioural performance: in the mental rotation task reaction times included more extreme values and were more variable compared to the flanker task. In summary, it appears as if (at least in the conducted tasks), both age groups could be forced to comparable performance levels with respect to error rate, while the older subjects still responded slower than the young. This is in line with the results of Eppinger et al. [13], at least with the behavioural results.

Despite this equal accuracy (i.e. comparable error rates), the psychophysiological data showed considerable differences between both groups. The Ne was consistently smaller for the older than the younger subjects. Moreover, the analysis of the variability of the Ne between trials, and the results of the independent component analysis implicate that the Ne of both groups is not fully comparable in the standard ERP analysis: it is more variable for older subjects than for the younger subjects. This variability was not reflected in the behavioural data.

Also, though the manipulation of the stimulus response mapping (or task difficulty), did have an impact on the Ne by means of a main effect of age group and task, this did not go in line with the behavioural results. Thus, the question about the function of the Ne arises in general. It might be that the amplitude does not play a crucial role for general performance, though it is obviously related to learning. Though this is in line with previous work of Masaki et al [27], who also concluded that the amplitude of the Ne does not play a crucial role for the effectiveness of the function linked to the Ne, this point has to be object of further investigation. Also the results are not in line with the results of Mathewson et al [28] who showed that the Ne was sensitive to age, but not to task demands. In the present study the Ne was clearly sensitive to both, task demands and age.

Nevertheless, in previous studies it has been shown, that the amplitude of the Ne is linked to learning [29], and there is a close interaction of learning success, Ne amplitude and aging [12], [13], [30].

However, these studies only partially explain the Ne effect in tasks which typically provoke erroneous reaction “slips”. Hence, in that context one explanation might be that the task was more difficult to learn for the older. Thus, their Ne is smaller compared to the young subjects. However, in the present study, an adaptive deadline with respect to the error rate was applied, thus both groups showed comparable error rates *during* the experiment and overall. Anyhow, it cannot be excluded that both groups show different learning rates, i.e. one group reaches the adaptive criterion earlier than the other group. This would require to keep the response deadline constant for both groups. However, the utilized tasks herein are not comparable to learning tasks since the flanker and the rotation task typically provoke reaction slips, whereas learning tasks initially, i.e. at the start of the experiment, provoke mistakes. This is an important distinction, since the interpretation of the Ne-effects depends on the utilized paradigm.

Furthermore, these results of learning experiments are supported from results of tasks with greater deadlines. Here older show a greater variability, whereas the source of this greater variability is based on performance during a brief initial exposure to an experimental task during which performance is improving more rapidly with practice for older than for young subjects (e.g., [21], [26]). Thus, it is possible that the greater variability reported for older adults may simply reflect the fact that their RTs improve more with practice. In the present tasks an adaptive deadline was utilized which might have forced both groups to a comparable performance improvement. However, this hypothesis has yet to be tested.

Another reason for the Ne effect might be that a task with a very strong stimulus response mapping might be much easier for young subjects compared to older subjects and that the Ne is also related to task difficulty. Here, it has to be distinguished between objective and subjective task difficulty. It has already been shown, that the Ne is attenuated for undetected errors [31]. Hence, it should be tested whether older subjects differ with respect to error detection. Furthermore, it might be that one perceives a task to be more difficult, but does not commit more errors. With respect to the present study, it would be interesting for further investigations to assess perceived task difficulty for different age groups.

Also, it has been shown that the flanker task automatically activates corresponding responses [32]. This automatic activation could be altered in higher age per se, which might be a confound.

Thus, one would predict that the decreased Ne is a result of task difficulty and stimulus response mapping, rather than age. For this purpose we conducted the mental rotation task, which is not only more difficult than the flanker task, but also has a much weaker stimulus response mapping. Apart from the expected main effect of task on the Ne amplitude the results show again a main effect of age, namely a smaller Ne of older.

Interestingly, the component structure by means of independent components of the EEG, as well as the variability of the Ne between trials differed considerable between both groups and tasks. The Ne-cluster explained less variance of the grand average ERPs in older subjects compared to young subjects. Also the Ne of older was more variable than the Ne of young participants. However, this increased variability was not correlated with behavioural variability. Obviously, the elderly were able to conduct both tasks with the same accuracy like young participants anyway. Thus, the question of the functional role of the Ne in general arises.

It can be shown, that the latency of the Ne predicts corrective behaviour in the actual trial [5], [33] and slowing in the following trial (e.g., [4], [32], [34], [35]). If the amplitude of the Ne is considered to be a correlate of cognitive control (in terms of a positive correlation), the declined Ne in elderly would be interpreted in such way, that cognitive control is declined in elderly. However, this is not reflected by the data presented here. Both groups were forced to comparable performances by means of error rates, and within both groups a balanced speed-accuracy instruction should compensate for trade-offs. Thus, the differences between old and young were not due to speed accuracy trade –off. The remaining RT differences did not correlate with the psychophysiological data. Though both groups had comparable coefficients of variation of response times, they did differ with respect to the variability of the Ne between trials. Also this variability was uncorrelated with ex-Gaussian parameters like tau and sigma. Thus, it appears as if the older participants did not show less control than the younger ones. Indeed, they could be forced by instruction and adaptive deadline to comparable performance, at least with respect to error rates. This performance did not correlate with the component structure (by means of ICA data) or, the variability of the Ne (by means of PCA of single-trials Ne).

In summary, it can be concluded that [a] the Ne (as a standard ERP) is not fully comparable between older and subjects, [b] despite comparable reaction times with respect to variation and outliers, the Ne is more variable in older subjects, thus [c] thus it might be that elderly compensate decreased control by some up to now unknown mechanism.

## Materials and Methods

### Ethics statement

The study was conducted according to the code of ethics of the World Medical Association (Declaration of Helsinki) and was approved by the ethics committee of the Leibniz Research Centre of Working Environment and Human Factors at the University of Dortmund. All data were analyzed anonymously. All participants gave written informed consent prior to participation.

### Participants

A sample of 36 healthy subjects participated: 20 young subjects (range = 20–30, m = 24.05, sd = 2.03) and 16 older subjects (range = 55–65 years, m = 60.56, sd = 3.86). All gave written informed consent prior to participation and received 10,- €/h payment. Groups were matched with respect to duration of education and socio-economic status. Both groups conducted digit-symbol tests [36] (HAWIE-R subscale) and MWT-B tests [37]. Age was significantly correlated with MWT-B performance (r = .52, t = 3.67, df  = 34, p<.001), but not with the digit symbol scale (r = −.1, t = −0.73, df = 34, p = .46).

### Experimental Design and data acquisition

In order to control for a confound of response strategies and age (i.e. speed and accuracy) the experimental design consisted of a mixed 2×2×2 design with the between groups factors age (young, older) and instruction (speed, accuracy) and the within subjects factor task (flanker, rotation). The factor instruction was nested within the factor age yielding 4 experimental groups. In the speed instructed group subjects were instructed to respond as fast as possible. In the accuracy instructed group subjects were instructed to respond as accurately as possible.

Participants were seated in an ergonomic seat in front of a 19″-CRT monitor (100 Hz). Responses were given by a button press of the left or right thumb. Each task consisted of eight blocks (one training block) with 80 trials each. Following each block a break of 20 sec was provided. After half of the experimental blocks a break of 120 seconds was provided.

The first task was a modified flanker task [38]. In the center of the screen an arrowhead indicated the button which had to be pressed. Hence, the stimulus-response mapping was very direct in the flanker task. This arrowhead was accompanied by two distracting arrowheads below and above which appeared 100 ms prior to target occurrence which is known to induce maximal distraction [31], [39]. These flankers could be congruent (pointing to the same direction) or incongruent (opposite direction). The occurrence of congruent and incongruent flankers was equiprobable.

Since, the flanker in the task as introduced by Kopp et al. [38] activates responses by symbolic spatial information [i.e. the arrows point to one direction], which is indicated by alterations of event-related lateralizations of the EEG over sensory and primary motor areas (as a lateralized readiness potential) [32] and this might be confounded with age, a second task with weaker S-R mapping was conducted. This second task was a mental rotation task modified for ERP measurement to yield a comparable time line and workflow to the flanker task for the participants during conduction of the experiment. One out of two letters (F,R) was presented to the participants. This letter was either rotated, mirrored across the main axis or both. Subjects had to indicate with a left or right button press of the corresponding thumb if the letter was mirrored or not. The letters were rotated by 0°, 45°,135°, 225° or 315°. The 20 possible stimuli (5×2×2) were presented in random order. Thus, the rotation task was not only much more difficult than the flanker task; it also differed with respect to the degree of stimulus-response mapping, which was quite indirect.

In each trial Subjects received feedback indicating whether they responded fast enough or too fast/too slow. The feedback consisted of two pictograms. If the participants responded fast enough a yellow pictogram of smiling face (“smiley”) appeared in the center of the screen. A red angry looking pictogram (“frowney”) appeared if they responded too fast or too slow. To exclude that any differences between both age groups are due to Speed-Accuracy trade-off, both age groups were nested in a between subjects design with accuracy or speed instruction. The accuracy instructed group had to respond as precise as possible whereas the speed instructed group had to respond as fast as possible. To stress the instruction, both instruction groups received feedback of their performance (Accuracy: *‘You committed x % errors during the last block.”*; Speed: *“Your reaction time was x ms in the last block.’*) following each block.

An adaptive deadline was applied in order to force both groups to comparable error rates. The deadline for the feedback was adapted block wise. If the error rate in one block (80 trials) was below eight percent, the deadline was decreased adding one standard deviation to the mean RT in the previous block. If the error rate was above 12% the deadline was increased by adding four standard deviations to the mean RT of the previous block. The purpose of the adaptive deadline was to prevent speed-accuracy trade-off. It could be that older subjects respond more slowly, but more precise. Thus, it was desirable to keep the error rate constant across groups, irrespective of their instructed response strategy.

EEG was recorded unipolar from 55-standard electrode positions with a sampling rate of 500 Hz. The EOG was recorded from the outer canthi and from above and below the right eye (SO2, IO2, LO1, LO2). Impedances were maintained below 5 kΩ. Data were re-referenced off-line relative to average reference.

### Analysis of behavioural data

With respect to behavioral data mean response times and error rates were analyzed by means of mixed effects ANOVAs (age group x task x response). The instruction factor was skipped for further analysis of behavioral as well as EEG-data, since ANOVAs revealed no substantial effects (i.e. significant), which is likely due to a lack of statistical power in this comparison and due to the deadline adapted to the error rate, thus it is not surprising that here is no effect of the instruction (what was intended). In addition, ex-Gaussians and the coefficient of variation of response time were calculated. Ex-Gaussians were fit to the response time data, since the shape of response time distributions might be altered in elderly subjects. It is a common result that elderly show more behavioral variability. Since the Ne is a response related potential, it might be that the Ne-amplitude effects are due to a higher response variability of the older group. Thus, it can predicted that higher response variability is accompanied by a higher variability of the Ne-amplitude. The ex-Gaussian is a mathematical model used to describe response time distributions. Ex-Gaussian functions provide good fits of empirical response time distributions and have been widely adopted (e.g., [24]). Briefly, the ex-Gaussian distribution is a convolution of a Gaussian and an exponential distribution, and it has three parameters: µ, σ, and τ. The latter reflects both the mean and standard deviation of the exponential portion, whereas µ and σ represent the mean and standard deviation of the Gaussian portion respectively. Ex-Gaussian analyses allow differences between conditions to be separated into distributional shifting, reflected in µ, and distributional skewing, reflected in τ. This approach is more sensitive to group differences in RT distribution than the classical approach with Gaussian parameters only. In the present study ex-Gaussian parameters were estimated by minimization of the negative log likelihood function. Fit of the single subjects ex-Gaussians were tested by mean of non-parametric Kolmogorov-Smirnov tests and the parameters were tested by means of mixed effects ANOVAs (adjustment of the degrees of freedom was made if appropriate, effect sizes are reported by means of partial eta squared [η^2^]).

For each subject the coefficient of variation of the response time (cvRT) was calculated as the standard deviation divided by the mean RT within subject, and was taken as a measure of behavioral variability. The scaling procedure in cvRT minimizes differences between groups that might arise from differences in mean and standard deviations [40]. Only RTs within the limits of 100 to 1000 ms were used.

### Analysis of EEG-data

#### ERPs

EEG-data were analyzed by peak-analysis of ERPs, principal component analysis, and by means of independent component analysis. Initially EEG data were manually cleaned from artifacts and filtered offline using a short non-linear FIR filter (highpass 0.5 Hz, lowpass 25 Hz). Following initial artifact correction by ICA (see below) data were segmented relative to response execution (−200 ms: 700 ms). For quantification of the Ne-amplitude and to bypass the possibility of smearing of the ERP by single-trial latency jitter, the average single-trial amplitude of the Ne was computed. The single-trial peaks were quantified by the difference between the most positive peak in the time-window −80∶0 ms prior to response onset and the most negative deflection in the time-window 0∶150 ms. Statistical analysis was conducted utilizing linear mixed model ANOVAs (group x task). Adjustment of the degrees of freedom was made if appropriate and effects sizes are reported by means of partial eta squared (η^2^).

#### PCA

To receive an estimate of the Ne variability between trials, a PCA was calculated with the erroneous trials of the EEG data at electrode FCz. Each subject's data was divided into channel specific matrices of single trial data with trials as columns and time points as rows. The dimensionality of each matrix was determined as a minimum number of principal components capturing 90% of the variance across trials. The chosen time window for these single-trial signals was that of the Ne (40–120 ms following response).These single-trial matrices were submitted to a PCA. Subsequently, the Eigenvalues were utilized for estimating the percent of variance each PC was explaining for the variance between trials. Note that this measure gives an estimate of the averal signal variability across trials. Thus it does not provide information about the variabilty of the Ne amplitude or latency.: It integrates these information i a single dimensionality measure. This procedure was done for the concatenated error trials of each subject. The so derived numbers of components for each subject were analyzed by means of mixed effects ANOVAs [for a comparable analysis see [40]].

#### ICA

ICA was conducted with the unsegmented raw data with extended infomax [41], [42], [43].For this initial ICA the full component space for each data set was decomposed (i.e. 59 components. Since the EOG had the same reference as the EEG EOG channels were kept in the decomposition). Following this, artifact correction was conducted using the derived independent components, i.e. by removing sporadically occurring huge artifact activity in the continuous independent component activations. Utilizing the independent component activations for artifact correction is a useful preprocessing step, since huge and rarely occurring artifacts can be reliably identified in the component activations by the fact that they typically spread across all activations at the same time point. It is desirable to remove those activations since ICA (at least infomax) cannot deal with rarely occurring events, thus the decomposition might fail. Thus, initially utilizing ICA decomposition for artifact removal improves the stability of a second decomposition [44]. Subsequently, a second ICA was conducted on the so pruned data set with the full component space and a second artifact correction was conducted. The next analysis step was a dipole analysis to model the derived components by a spherical 3-d model. All components that could not be located within the cortex, components with dipole positions that were located with a higher residual variance than 15% as well as artifact components (eye movements, blinks, and muscle artifact) were removed, i.e. the data were cleaned by backprojection of the remaining components to the scalp [45]. Artifact components were identified visually by inspection of the pseudo-inverse of the weights matrices and components' time course. Note that this was combined with the 15% threshold for estimation of the sources of the IC topographies. On average 7–8 components were rejected for both age groups. For detecting the component accounting for the Ne activity being specific for error trials the mutual variance between each component and the Ne in the corresponding time window was calculated. This was done by estimating the percent of variance the components accounted for in the critical time window of Ne occurrence. Furthermore, it was manually checked whether these components varied with the erroneous response, showed a typical Ne-topography and a comparable dipole localization. Finally, cluster analysis (k-means) was conducted with the scalp topographies, ERPs and dipole localizations to validate the results of the manual component identification. To model the neural source of the remaining components the grand average IC-topography was analyzed by utilization of the DIPIFIT plug-in in EEGLAB [46]. This plug-in can be utilized to model neural sources of independent component scalp topographies by means of source localization by fitting an equivalent current dipole model using a non-linear optimization technique and a 4-shell spherical model [47], [48]. All analyses were conducted using Matlab©, EEGLAB and custom Matlab scripts. For statistical analyses GNU R [49] was utilized.
